# Community advisory committee as a facilitator of health and wellbeing: A qualitative study in informal settlements in Nairobi, Kenya

**DOI:** 10.3389/fpubh.2022.1047133

**Published:** 2023-01-09

**Authors:** Ivy Chumo, Caroline Kabaria, Clement Oduor, Christine Amondi, Ann Njeri, Blessing Mberu

**Affiliations:** African Population and Health Research Center (APHRC), Nairobi, Kenya

**Keywords:** community advisory committee, health and wellbeing, community advisory board (CAB), informal settlements, qualitative study, community based participatory research

## Abstract

**Introduction:**

A range of community engagement initiatives to advance health and wellbeing are currently taking place in informal settlements in low and middle income countries (LMICs), including community and stakeholder meetings, use of radio, film, TV programs and other information, education and communication materials (IECs) organized by different stakeholders. While these initiatives tend to focus on unidirectional flow of information to communities, the need to incorporate initiatives focusing on bi or multi-directional flow of information have been identified. Despite the extensive body of literature on community engagement, the role of Community Advisory Committees (CACs) in advancing health and wellbeing in informal settlements is still a puzzle, occasioned by considerable ambiguity. A community advisory committee is a dedicated group of volunteers to support health and wellbeing needs of their community using a community approach. Researchers and project implementers work in partnership with CACs to successfully implement their activities within the target community.

**Methods:**

In this paper, using in-depth interviews, we document the roles of CACs in advancing health and wellbeing in Korogocho and Viwandani informal settlements in Nairobi, Kenya.

**Results:**

Study participants described the role of CAC in advancing health and wellbeing through education and awareness creation, advisory roles in research and implementation goals, protecting community interests and acting as gatekeepers and collaborators to community partners. Identified barriers to achieving CAC roles include lack of finance and other field resources, being labeled as organization staff and low involvement by some upcoming and emerging local leaders on issues which involve the CAC constituents. Enablers of CACs in their roles include possession of appropriate skills and values by members; involvement of the community in the selection of members, regular consultative and advisory meetings, representativeness in the composition of CAC membership and knowledge about the community.

**Conclusion:**

We conclude that CACs play key roles in advancing health and wellbeing in informal settlements and that existing CACs mechanisms and operations need to be given due consideration by researchers, project implementers and local authorities right from project conceptualization. CACs need recognition beyond consultations and placations during research and project implementation to a veritable social structure for community's social viability and survival as well as partners in development for inclusive urbanization process. While CACs have contributed in advancing health and wellbeing in informal settlements, there is need for a long-term strategy to optimize their impact and reduce puzzles around their roles.

## Introduction

Globally, there is a universal agreement that health and wellbeing challenges in informal settlements are unacceptable and require urgent action (1, 2). Cities have unequal outcomes and opportunities because many population groups residing in informal settlements are systematically excluded from participating in health and wellbeing activities, processes and decisions (2, 3). Participation by study communities is important, as growth often occurs so quickly that urban planners do not know the actual population density and needs of residents in the informal settlements (4). A lack of community participation leads to a situation in which public resources fail to reach the vulnerable groups (5, 6), as such most residents of informal settlements face asymmetries in health and wellbeing advancement and opportunities in informal settlements (7). Past approaches toward the health and wellbeing asymmetries in informal settlements were characterized by a combination of patronage and neglect, insecurity and inequity (8). This was accompanied by a blinkered approach to data and knowledge of settlements (6, 9). Past 10 years have witnessed an intense focus on health and wellbeing aspects of land, housing, labor and basic amenities (10, 11). The approaches together accompanied by a denial of residents' participation have created conditions in which the settlements continue to be bundles of exclusions (10). Social exclusion is a determinant of health and wellbeing challenges in Africa's informal settlements, and can be reversed through social participation (12). Promoting social participatory mechanisms in Africa's informal settlements can be an efficient principal formula for advancing health and wellbeing (13). One principle mechanism that produces a relationship between social participation and advanced health and wellbeing is community engagement (8, 14).

Community engagement approaches promotes health and wellbeing by ensuring the involvement of communities as true partners in all phases of community activities based on key principles that include, amongst others, the recognition of the community as a unit, the use of community structures and strengths, and sharing access to information and knowledge with all partners (15, 16). However, community engagement process in Kenya's informal settlements does not always advance health and wellbeing, as there is minimal active engagement of community members as partners in all aspects of community activities (17). When partners are fully engaged, they contribute their expertise in understanding a given phenomenon and integrate rich local knowledge gained with action for advanced health and wellbeing related benefits to communities (14, 18). Over the last 15 years, there has been growing attention on the principle of establishing partnerships through community advisory committees (CACs) for active community engagement and success of project and research activities (18, 19). CACs are a dedicated group of volunteers who support health and wellbeing needs of their community using a community approach. Researchers and project implementers work with CACs for successful implementation of their activities in the best interest of the target community. The committee comprises representatives from community members, government authorities and service providers. The representatives jointly serve as liaison between the partners and the community, thus, curbing a one-way flow of information from the partners to communities (20, 21). Despite the extensive body of literature on community engagement, the role of CACs in advancing health and wellbeing in informal settlements is still a puzzle (18, 21), occasioned by considerable ambiguity (15).

It was with this background that we sought to deeply understand the roles of CACs as established in the two informal settlements where the African Population and Health Research Center (APHRC) runs the Nairobi Urban Health and Demographic Surveillance System (NUHDSS) in promoting health and wellbeing over time. NUHDSS was established by APHRC in 2002 to provide a platform for investigating the long-term social, economic and health consequences of urban slum residence, and to serve as a primary research tool for intervention and impact evaluation studies focusing on the needs of the urban poor (22). In addition to the routine data, NUHDSS has provided a robust platform for nesting several studies examining health and wellbeing challenges of rapid urbanization in Africa. The conceptualization of CAC began in 2012 as part of how the APHRC would work collaboratively with the study communities to contribute to research and implementation projects. Overtime, the CACs has been beneficial to researchers and implementers (i.e., from within and outside APHRC) while engaging with the study communities. Similarly, Accountability and Responsiveness in Informal Settlement (ARISE) project requires an active participation of the study communities (23) in evidence generation as co-researchers and in co-creation of solutions to community challenges. As a result, the project team, at its inception, identified the need to assess and document the roles of CACs so as to facilitate community engagement. This becomes important as diverse influences have resulted in considerable ambiguity about the current roles of CACs (20). Consequently, this paper seeks to address the ambiguity and knowledge gap, by documenting the roles of CACs in advancing health and wellbeing in Korogocho and Viwandani informal settlements in Nairobi, Kenya.

## Methodology

### Study design

This was a qualitative study using in-depth interviews (IDIs). As an exploratory and descriptive study, IDIs have the potential to generate more insightful responses from those who know about the community. With none of the potential distractions or peer-pressure dynamics that can sometimes emerge in focus groups, IDIs provide insightful and possibilities for high valuable findings, with interviewers having greater opportunities to ask follow-up questions, probe for additional information, and circle back to key questions later on in the interview, which will ultimately generate a rich understanding of attitudes, perceptions, and motivations (24) in our case on the roles of CAC in both communities.

### Study setting

**Figure 1:**
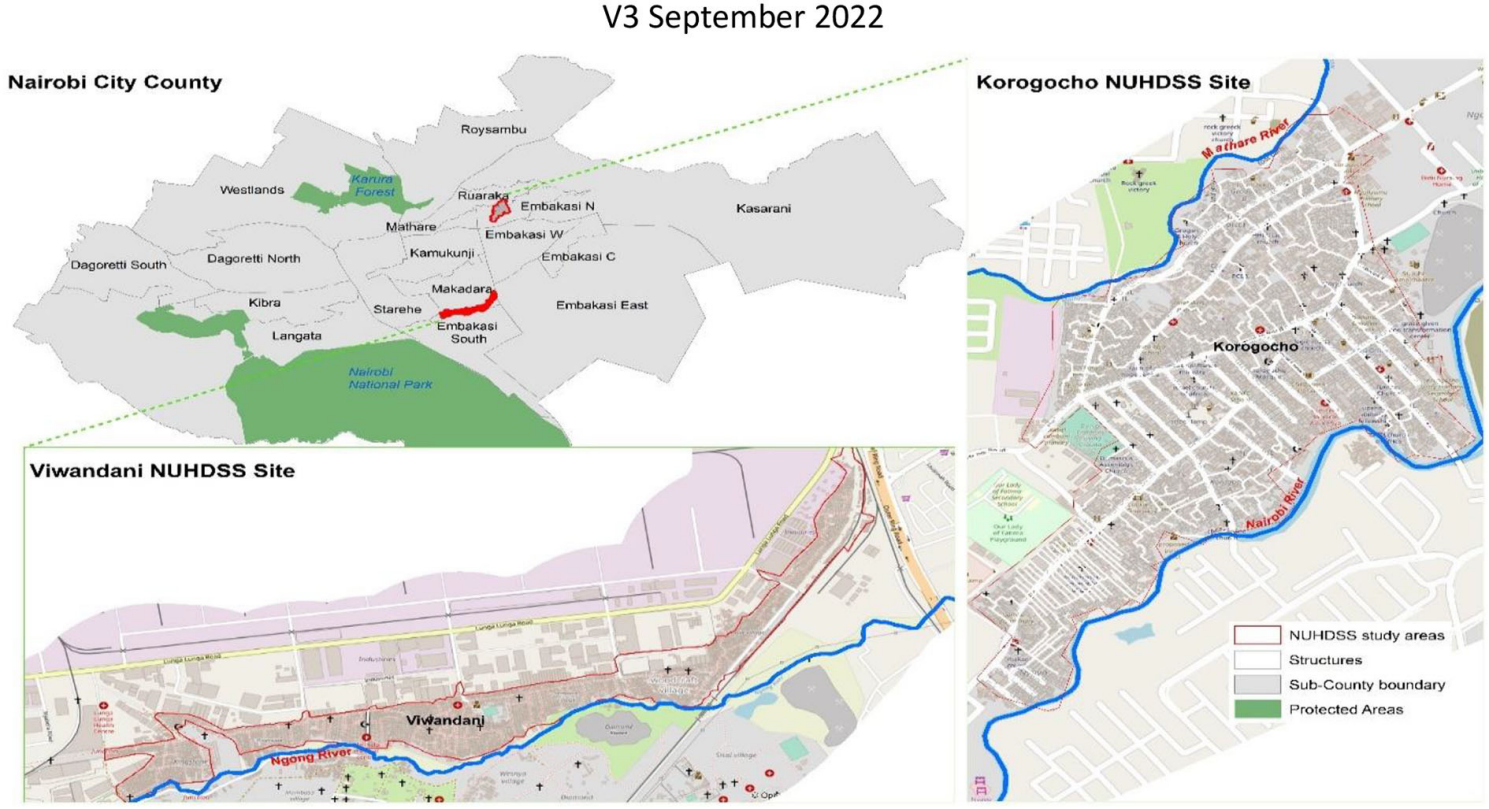
Study sites (Source, authors, 2020).

The study was conducted in Korogocho and Viwandani informal settlements in Nairobi (Figure 1), in the areas covered by NUHDSS initiated in 2002 by the APHRC (22). Korogocho has a stable and settled population and residents have lived in the area for many years (25), while Viwandani is located next to an industrial area with many highly mobile population who work or seek jobs in the industrial area (25). The community advisory committee working in each of the NUHDSS sites is composed of approximately 13 members, who were elected by respective constituent groups that they represent. The members represent various constituencies in the community, government, local leaders/village elders, the youth, women, older persons, school administrators, healthcare providers, Faith-based organizations/Community based organizations/Local Non-governmental organizations (FBOs/CBOs/local NGOs), Community Health Volunteers (CHVs), media/education and entertainment organizations, religious groups and people living with disabilities.

### Target population, sampling and sample size

The population of interest were CAC members, community members, community-based organizations (CBOs), Researchers and Implementers and Community Liaison persons. The population of interest ought to have interacted or heard about CAC. The study population allowed for data triangulation and were drawn from APHRC, the organization that facilitated the formation of the two CACs in Korogocho and Viwandani informal settlements in Nairobi, Kenya. Data was collected from all 13 CAC members in each study site as they represent different sectors/constituencies in the community. Data collection tools were also administered to two Community liaisons and six Researchers and implementers who worked closely with CAC in the study sites. Lastly, the tools were administered to five community members and four CBOs in each study site, who were selected if they had interacted or knew about CACs.

### Data collection process and data quality

We collected data from 19/05/2020 to 14/06/2020. Research Assistants were selected if they had been endorsed by community leaders to be trusted community residents in the study sites and by the researchers if they generally had at least 5 years of experience in qualitative research. Research Assistants were trained for 5 days on the aims of the study, data collection process, data collection tools, and research ethics. Pretesting was done with one former CAC member and two community members to establish the length of the interviews and to ensure the language could be understood by the study participants. Data was collected through In-depth Interviews (IDIs), using an IDI guide which had questions on the role of CAC in advancing health and wellbeing. The interviews were conducted in English and Swahili and were held through a mix of face-to-face and phone interviews at a quiet location convenient to the participants which were mainly at their homes and sometimes remotely through phone calls at the convenience of the study participants. The interviews were recorded using a digital recorder and backed up with handwritten notes. These interviews lasted for ~1 h.

We reviewed all audio files to ensure completeness and depth of the interviews, and provided feedback to the field team at the end of each working day. Debriefing sessions were also held at the end of each working day to highlight the key emerging themes, review probing techniques, and assess progress.

### Data analysis

Transcripts were imported into NVivo 12 software (QSR International, Australia) for coding and analysis. NVivo is a qualitative data management software that can be shared and worked on in groups and facilitate thinking, linking, writing, modeling and graphing in ways that go beyond a simple dependence on coding (26). We used a framework analysis (27). Framework analysis is adopted for research that has specific questions, a pre-designed sample and priory issues (27). The first step of framework analysis was listening to the recordings to familiarize the researchers with the information related to role of CACs. To ensure reliability, two researchers (an experienced qualitative researcher with community engagement and an anthropologist) and five co-researchers, who collected the data participated in the development of a coding framework by reading the outputs imported in NVivo 12 software independently to establish an inter-coder agreement. Once the initial coding framework was completed, the team met to discuss the themes generated and to reach an agreement on themes. Two researchers proceeded with coding, charting, mapping and interpretation of transcripts. Similar emerging issues were combined into single categories through consensus discussions.

### Ethical considerations

The study was approved by the AMREF Health Africa's Ethics & Scientific Review Committee (ESRC), REF: AMREF-ESRC P747/2020. We also obtained approvals from National Commission for Science, Technology and Innovation (NACOSTI), REF: NACOSTI/P/20/7726. Approval was also obtained from the Liverpool School of Tropical Medicine (LSTM) and the African Population and Health Research Center (APHRC) internal ethical review committees. All participants provided informed written consent before participating in an interview. The interviews were conducted in quiet spaces for privacy, confidentiality and for the quality of the audio files. All participants provided informed written consent before participating in an interview, and were free to drop from the interview at any point in time. The interviews discussions were anticipated from the onset to take ~ 45–60 min, as such we compensated the study participants for their time.

## Results

### Demographic characteristics of the study participants

We present the demographic information of the study participants by category and; by gender in Table 1. These demographics were largely similar in Korogocho and Viwandani.

**Table 1:** Demographic characteristics.

**Participant category**	**Gender**		**Total**
	**Males**	**Females**	
**CAC**			
Community health volunteer (CHV)	1	1	2
Older persons representative	1	1	2
Women representative	1	1	2
Community members	2	3	5
Community-based organizations CBOs	2	2	4
Researchers	X	Y	3
Implementers	X	Y	3
Community liason	1	1	2
Total	X	Y	52

We present our findings following the core theme of the role of CAC in advancing health and wellbeing, and emerging themes of enabling factors and barriers to achieving the roles. The themes and corresponding subthemes that emerged from our analysis are summarized in Table 2 that follows.

**Table 2:** Study themes and subthemes.

**Themes**	**Subthemes**
Role of CACs in advancing health and wellbeing	• Education and awareness creation • Advisory roles • Advance research and implementation goals • Protecting community interests • Gatekeepers and partners
Enabling factors in achieving the role of CACs	• Community values • Intrapersonal and interpersonal skills acceptable to the community • Knowledgeable about the community • Governance structure of CAC
Barriers to achieving the role of CACs	• Lack of finance and other field resources • Low empowerment to perform duties and being labeled as organization staff • Limited time allocated for meetings • Challenging emotional reports

#### Themes 1: Role of CACs in advancing health and wellbeing

Study participants described that the roles of CACs in advancing health and wellbeing included education and creation of awareness, advisory role, advancing research and implementation goals, protecting the interest of the community and acting as gatekeepers. The roles were interconnected, for example in the process of creating awareness, CAC could simultaneously be protecting the interest of the community.

##### Education and awareness creation

Study participants described CACs as playing a key role in educating the community on health and wellbeing issues, through consistently creating awareness in the community on the same. The education and awareness creation was linked to their attempts to narrow service delivery gaps in water, sanitation, hygiene, use of face masks in the phase of the Coronavirus disease (COVID-19) pandemic, general cleanliness, maternal and; child health and security.

“*We educate people… I have to talk to every person I meet and tell them that they must boil or treat water before drinking… we ask them to clean their toilets and use the toilets accordingly because if that is not done, people become sickly*.” (IDI, CAC, Korogocho, Female).

To reach a vast population, CAC members utilized existing social structures and networks. These included community meetings, social gatherings, support group meetings, and public transportation.

“*During church meetings and other social gatherings, we educate the community on the importance of hand hygiene, drinking of clean water and on the importance of NHIF {medical insurance} cards… We create awareness on wearing masks, even while on public transport or in other forums”* (IDI, CAC, Korogocho, Female).

Study participants described how CAC empowered the community on how to ensure that the researchers/partners gives back to the community. While creating awareness, CAC was also described in terms of relaying the right information to the community in the right way.

“*We educate people to ensure that schools have kept the environment clean, children are not harmed, and the school environment is good for the children; we bring good coordination between the parents, teachers and the children… We {CAC} ensure the information needed to reach the community gets to the community as needed and in good time”* (IDI, CAC, Viwandani, Male).

##### Advisory roles

Study participants agreed that CACs play health and wellbeing advisory roles to stakeholders in the community. The advisory roles described ranged from the inception to completion period of a project or program. Respondents stated that while implementers/researchers were giving input during planning and protocol development, the views of lay community members on projects ought to be respected. In their advisory role, CACs allowed for debates with the implementers/researchers for mutual agreement with the community thus advancing health and wellbeing.

“*We advise partners; researchers, implementers or even community members from the beginning to the end… then depending on their capability, they can follow our advice on when, where and how a program or project could be conducted. If they are not able to follow our advice, we discuss it further and give room for debate. We cannot send anyone away”* (IDI, CAC, Korogocho, Male).

Researchers also confirmed what the CAC described on how they play a great advisory role:

“*We always seek advice from CAC before doing any community activity including research… sometimes in the course of a one-year project, we meet with them up to 6 times for advice on how to conduct research or implementation activities at the community and who to involve in implement projects and what would be the best time to conduct research and implementation activities”* (IDI, Researcher, Male).

The advisory roles were described to go beyond the research and implementation team to the other stakeholders in the community including community members, CBOs and service providers. As such, CAC was acknowledged by the community and community-based organizations to be playing an important role in advancing health and wellbeing.

“*When we suspect that the vulnerable people in the community like the persons with disability are mistreated by some partners in the activities they do at the community, we usually consult and seek advice from CAC… We do interact a lot and I think the administration relies a lot on us for our advice on community issues, more so to ensure no one is discriminated against by anyone”* (IDI, Community member, Korogocho, Female).

“*We {CBOs} normally have an interactive discussion with our partners about specific health and wellbeing services, we usually invite some CAC members for advice on how to bring them closer to the community”* (IDI, CBO, Viwandani, Male).

##### Advance research and implementation goals

Study participants described how advancing research and implementation goals was a primary role of the CACs in their attempt to advance health and wellbeing. The participants consistently stated the roles of CAC included identification of household structures and study participants for a research or an implementation program. Participants' interpretation of these roles included examining issues of consent and confidentiality, coercion, support, community involvement and acting as liaison in research and project implementation.

“*We ensure that researchers and implementers observe privacy and do not disclose personal issues to others without the owner of information being notified. We also ensure that the community is well informed and involved without discrimination by anyone and that no community member is forced by implementers or researchers to be part of a program”* (IDI, CAC, Korogocho, Female).

One of the researchers said the following about the CAC's insights about the study communities and how these insights help inform the researchers' understanding in the process of implementing their studies:

“*They [CAC] know their communities better than we do. They work with us in implementing our studies and to help us understand the community”* (KII, Researcher, Male).

While advancing research and implementation goals, CAC was also described as playing a two-way mutually informative liaison role between the community and the researchers and project implementers during the research and project implementation phase. For example, study participants described how the community members could easily access CAC to relay information to researchers and implementers and on the other hand, researchers and implementers could easily access the CAC to relay information to the community during a project implementation phase. As such, researches, implementers and community members trusted CACs with problem resolution.

“*CAC have a great understanding of the community. If there are problems with stakeholders in the community, CAC assists with resolving them.”* IDI, CAC, Viwandani, Female).

“*The role of CAC is to be a link between project implementers or researchers and the communities and to feedback to the researchers and implementers on issues”* (KII, Community Liason, Female).

##### Protecting community interests

Our study revealed how CAC members protected community interests by representing the community and providing substantive input into a project implemented at the community. As such, study participants described how CAC represent the community in resolving community issues related to security, education, sanitation, water and health among others.

“*We are always at the forefront in everything that happens here in the community in the attempt to ensure that the community benefits. We do this by providing input into different projects. We do help both the researchers and the community*. W*e discuss many issues in our meetings for the benefit of the community”* (IDI, CAC, Korogocho, Male).

Participants described CAC as an eye on what happens in the community, while protecting interest of the community. There were other discussions on the role played by CAC in assessing and monitoring the integrity of community activities and CBOs for the benefit of the community as a whole.

“*When research is being conducted, we are the eyes… If one wants to sponsor matters like health or water or sanitation, as the eyes of the community, we allow them to proceed if they are beneficial to the community… We assess the research and implementation projects to establish if they have truly come to help people in the community as they purport.”* (IDI, CAC, Viwandani, Male).

Researchers, Community members and CBOs description of how the role of CACs concurred with the roles in protecting interests mentioned by the CAC.

“*The primary role of CAC is to ensure there is community involvement on project activities for the benefit of the community”* (IDI, Researcher, Female).

##### Gatekeepers and partners

Study participants described how CAC members acted as gatekeepers and partners to stakeholders from outside the community. Before any individual gets to implement a project in the community, CAC members were involved. During project implementation, the CAC also acted as partners to the stakeholders and contributed to project activities.

“*We ensure that no one gets to the community without going through us… we act like gatekeepers and partners once the visitors or outsiders are in the community”* (IDI, CAC, Korogocho, Female).

Community members, researchers and CBOs also described CAC as gatekeepers, peacekeepers, and community representatives, who acted as partners for improved health and wellbeing and community functioning.

“*They {CAC} are like the gatekeepers in the community… CAC seek ways to sort the issues at the community for everyone”* (IDI, CBO, Korogocho, Female). “*CAC members are representing community for health and wellbeing equity in the community”* (IDI, Researcher, Male).

“*To the community, CAC is a peacekeepers; mediates for peace between community and new stakeholders… We {community} understand CAC members as people who represent us in many forums”* (IDI, Viwandani, Community member, Male).

#### Theme 2: Enabling factors in achieving the role of CACs

Study participants described enabling factors for the role of CAC and it included possession of community values, intrapersonal and interpersonal skills acceptable to the community, being knowledgeable about the community, and good governance structure of CACs.

##### Community values

Study participants described how community values enhanced the role of CAC. The CAC members had the following values acceptable by community, love for community, patience and being role models. The values enhanced trust and acceptance of the committee by the wider community. This promoted good relationships with people in the community and advanced health and wellbeing.

“*CAC members are volunteers and love the community… We are not paid a salary but we love our community. How well you show love and are patient to people is important because it promotes trust and acceptance by the community.”* (IDI, CAC, Viwandani, Female).

“*You must be a good role model, and active in the community… I usually tell the landlord and other tenants, 'Before one rent any of these houses, you must find out where he/she is coming from… and if you can't talk to that new tenant yourselves, please look for me so that I can come and find out where one is coming from'. This has really helped in promoting trust”* (IDI, CAC, Korogocho, Male).

##### Intrapersonal and interpersonal skills acceptable to the community

Study participants described how interpersonal and intrapersonal skills were enablers of the roles of CACs. The skills included flexibility, teamwork, leadership and passion. The skills enhanced the role of CAC members in their attempt to advance health and wellbeing in the community.

“*You must be flexible, team player and able to interact well for you to serve the role of CAC effectively… one who is disciplined should be a CAC member”* (IDI, Community member, Korogocho, Male).

“*Someone should have leadership skills and not be greedy but they should be looking for the interest of the community. For example, a CAC woman representative must be passionate about women and have women issue at heart.”* (IDI, CAC, Viwandani, Female).

##### Knowledgeable about the community

One ought to be knowledgeable about the community and the community constituency they represent. Study participants described how being knowledgeable enabled CAC members to perform their roles effectively.

“*A knowledgeable persons to improve a community are chosen to be in the CAC… If one has good community knowledge, they can be effective… I may be well educated but if I do not understand the problems of the community then I cannot serve well in the CAC”* (IDI, CAC, Viwandani, Male).

##### Governance structure of CAC

CACs governance structure provided for the community, through the various community constituencies to each build consensus on who may best represent their interest from amongst a set of volunteers from their own constituency. Furthermore, it allows for the CAC members to on their own agree on and set their routine consultative and advisory meetings within the year. As such, CAC members were seconded by their respective constituencies on the basis of their capacity to effectively represent their constituency, which is dependent on the residency or working in the informal settlement and knowledge of the issues that are important to their constituency and knowledge of the informal settlement.

“*Members are chosen by the community and in particular the members of a constituency. One of the requirements is that you must come from the community, the second thing you must have been working in the community as well as part of the role you are chosen, for example, a representative of health should be working in a health sector/role*” (IDI, CAC Korogocho, Male).

Study participants emphasized how meetings held regularly enhanced the role of CAC. Occasionally, service providers were invited to CAC meetings for debate on a common topic. This allowed for demanding equitable service provision on behalf of the community and their constituency.

“*There are times when we are brought together with education or hospital owners. There we brig the accusations before them. We tell them, ‘At your place, there was a client who was treated unfairly' this is actually how the service providers are compelled to offer the best so that they are not embarrassed in other meeting”* (IDI, CAC, Korogocho, Male).

CAC governance structure had guidelines, as such there were agreed measures and principles for ensuring adherence by the members to keep the CAC functioning. For example, CAC members who consecutively miss routine meetings without apologies would be discontinued from membership and the affected constituencies advised to provide replacements. CAC was described to be non-political but goal oriented.

“*We usually discontinue members who miss out on meetings without apologies… It is a committee that is not political… it is a committee that has principles and is focus oriented…”* (IDI, CAC, Viwandani, Female).

Notably, there were no reported cases of disagreements amongst CAC members as the CAC structure ensured that a member first and foremost represents the voice of their constituency, and not self. For example, a woman representative would be keen to represent issues of women in terms of how the proposed research topic or project activity would affect or impact them.

“*We have not had any conflicts so far because we represent different and separate groups. In the event that there is a conflict, we usually select a team lead and so he/she will lead in the resolution”* (IDI, CAC, Viwandani, Female).

Study participants described how CAC governance had a structured leadership that could help address any potential conflicts. For example, the leadership is composed of a CAC patron and the Area Chief, who is the representative of the National Government in Kenya and who sits in all the CAC meetings to help moderate conflict situations alongside the CAC Chairperson, usually elected by the CAC members as their leader.

“*CAC composition has got a clear leadership that enables prevention of conflicts. There is a patron for CAC, Area Chief and village heads, who oversee resolution of conflicts, in case it arise. However, we have had no conflict because we are interested in the community benefiting*” (IDI, CAC, Korogocho, Male).

#### Theme 3: Barriers to achieving the role of CACs

There were barriers to CAC effective operations reported which included: lack of finance and other field resources, being labeled as organization staff, low involvement by some local leaders on issues which involve their respective constituency and less time allocated for meetings.

##### Lack of finance and other field resources

CAC members stated that they did not have a budget allocation to support their roles in the community. The members did not have facilitation for field resources and communication, which made it extremely challenging to pass on information when needed.

“*At times during the rainy season, when we want to go on the ground, the lack of things like boots are a problem… We also need things like, bags and umbrellas.”* (IDI, CAC, Korogocho, Male).

“*Some things happen in the community that needs our service and our support but we do not have a facilitation for transport and communication*” (IDI, CAC, Viwandani, Female).

##### Low empowerment to perform duties and being labeled as organization staff

Despite that collective awareness creation and advocacy on CACs at the community, CAC members described how they were labeled as organization staff by the community, which made some of the members to feel less empowered in performing some tasks because community members expected too much from them. The labeling also sometimes made them felt left out from other important activities in the community that concerns their constituency, more so by upcoming influential groups at the community.

“*Some community members label us as staff of organizations who come to the community, so they expect much help than we can offer*… *We are not empowered. We cannot go ahead and do things out of our own volition. I represent the community but when it comes to issues that concern me and the constituency, I am not always involved by some local influential leaders…”*(IDI, CAC, Viwandani, Male).

##### Limited time allocated for meetings

CAC members thought that the frequency and time allocated for meetings in many instances did not allow in-depth assessment of issues.

“*We hold few meetings that last for an average of 2-3 hours only… Researchers and implementers have many other things to do and so they only allocate less time”* (IDI, CAC, Viwandani, Female).

##### Challenging emotional reports

Furthermore, CAC members mentioned that listening to issues from community members sometimes affected them emotionally, especially since they had a role to provide support to the person confiding in them.

“*Some issues brought to me by the community are challenging and you can feel like crying, but then you are supposed to be strong to keep the discussions”* (IDI, CAC, Korogocho, Female).

## Discussion

Our findings contribute to the literature on the role of dimensions of local social structures like CACs in advancing the viability of urban living in the face of increasing urbanization of poverty, especially among residents in informal settlements, building on partnership approaches in community engagement in promoting health and wellbeing. This is similar to other studies depicting the basis of CACs underpinned in the community development and public health needs to consistently and constantly engage the community (14). Our findings showed that turning the tide is not only possible, but it is also imperative, as many individuals working in informal settlements embraced the roles of CACs in advancing health and wellbeing. We describe how advancing health and wellbeing by reaching the poorest and most marginalized groups, requires strong community ownership of the value and purpose through for example working with CACs in informal settlements. We find specifically that CACs advanced health and wellbeing through education and awareness creation, advancing research and project implementation goals, advisory role, protecting interest of community and acting as partners and gatekeepers. These findings are consistent with other studies that have described how there is a clear urgency to advocate for reduced health and wellbeing challenges in informal settlements through community engagement (18). This follows an analysis of current trends, which provides evidence of persistent health and wellbeing challenges, yet, there is optimism and always a way forward through community adaptive mechanisms (8). While our findings do not show any reports of how differences amongst the CAC members could impede research or project implementation within the community, we note that the CAC governance structure provided for a leadership that could help address such differences before they get out of hand. For example, there is the CAC patron, the Area Chief, who is the representative of the National Government in Kenya and who sits in all the CAC meetings to help moderate in such situations alongside the CAC Chairperson, usually elected by the CAC members as their leader.

Noted enabling factors for the roles of CACs included possession of community values, intrapersonal and interpersonal skills acceptable to the community, being knowledgeable about the community, and good governance structures of CACs. The governance structure allowed for the involvement of the community in the selection of CAC members; convening consultative and advisory meetings, representative composition of members and a great knowledge of the community by CAC members. These factors enhanced the level of trust and acceptance of the committee by the wider community. This relates to other studies and experiences by CACs in South Africa (28) and Zimbabwe (19), describing enabling factors as involving the community, knowledge and understanding of the community and good CAC engagement structures (18). CAC members were volunteers that is, they offered themselves to represent their constituencies without any wages except a small non-compulsory facilitative allowance to partly enable them to discharge their various roles for the benefit of community, and as such motivation strategies are important for the CAC members to continue volunteering. Strategies that instill a sense of empowerment and capacity building are essential to promote the role of CAC members. This implies that there is a need to adopt recognition of CAC members' contributions of time, resources, and expertise, through some type of compensation and acknowledgment (29, 30). Many partnerships do not have the means to provide monetary remuneration (18, 19, 31). As such, partners, including implementers and researchers and NGOs should identify other means to promote CAC membership retention and ensuring that the benefits of membership outweigh the costs. Such strategies may include partners facilitating adequate orientation and training of new members, providing opportunities for social interaction, adequate access to information and resources, influence in decision making, and recognition for contributions. Inexpensive strategies to recognize members' contributions could include dinner parties, awards, certification, positive recommendation letters, and recognition in local media. Even though the provision of stipends or honoraria by the researchers/implementers in meetings (30) can serve as a form of recognition for the contribution of the CAC member, in resource limited communities even providing relatively small amounts of money in the form of transport reimbursements does have the potential for undue influence (19), as such it is important for partners to agree on an appropriate amount with the community and document an ideal compensation to be adhered to by every partner.

Our study showed that CACs played some roles that advanced health and wellbeing. However, engaging committees was described as time-consuming, as such meetings were felt not to exhaustively discuss all issues intended to be discussed by the CAC. This observation is consistent with other studies describing how time consideration for a meeting often go beyond what is usually perceived as directly related to the task/purpose of the research or implementation, to include providing transportation, technical assistance, and participating in community events (32, 33). This is important in addressing the opportunity costs of CACs membership and participation in its activities. Besides, there were challenges related to competing demands and financial and field resources, low empowerment to perform duties and being labeled as organization staff and challenging emotional reports. Other studies described how financial, political and institutional pressures make it difficult for individuals within these organizations to devote the requisite time, finance and energy to a particular community-based endeavor (34–36). Lack of financial, political and institutionalization could be the reason why the CACs faced the challenges. This implies that some CAC members would end up being less engaged at the committee during some seasons. This was supported by evidence from other studies describing how challenges faced by CAC members, led to some of them not being fully engaged when needed (21, 37–39). The challenging concerns do not negate the positive effects of the role of CACs but need identifying at an early stage during the planning of community engagement.

### Limitations of the study

Our study is not without limitations. The study was carried out during the peak of Covid-19 outbreak. However, we observed all the Covid-19 public health measures and successfully conducted the study. The study was also conducted in two informal settlements in Nairobi, Kenya. Korogocho study site represents settlements that are stable with a settled population, where residents have lived in an area for many years. Viwandani on the other hand represents settlements with many highly mobile population. The lessons may be transferable to other informal settlements, even outside of Kenya because of the diversity in informal settlements selected and groups and stakeholders represented.

## Conclusion

The past several decades have seen a resurgence of interest in partnership and participatory approaches to research and project implementation, with an emphasis on community engagement approaches that are beneficial to the communities. CACs provide a social infrastructure for community members to voice concerns and priorities that otherwise might not enter into the researchers' and program implementers' agenda and advise about suitable processes that are respectful of and acceptable to the community. It is fundamentally important that the CAC is able to carry out its functions independently of the research and implementation teams in order to protect the community from any unethical practices. A factor that has the potential to negatively influence this independence is the mechanisms for compensating CAC members for their time and providing resources for their functioning. As such, there is a need for researchers' and implementers' to adopt a long-term strategy to optimize the overall impact of CAC for the benefit of study communities.

We recommend that community engagement mechanisms like CACs should be of consideration to every research and implementation team during the conceptualization of a project, and not just for consultation and placation. This is because most people in participating communities beyond their low socioeconomic status, have a better understanding of their community needs as well as social norms than partners do. If the CAC initiative is strengthened and valued by community stakeholders, it will contribute to advanced health and wellbeing and lessen or do away with project/research implementation challenges in informal settlements in Kenya and beyond. There is therefore a need to adopt the recognition of CAC members' contributions of time, resources, and expertise, through some type of compensation. These calls for researchers and implementers to adopt innovative strategies to recognize members' contributions. In addition, it is important to identify other means to promote CACs' membership retention and ensuring that the benefits of membership outweigh the opportunity costs. Such approaches may include but not limited to adequate orientation and training of new members, creating opportunities for social interaction and participation, adequate access to information and resources, influence in decision making, and recognition of contributions of CAC members by the researchers and implementers. Our study findings implies that since compensation of CAC members by researchers/implementers may compromise their neutrality and ability to be the “gatekeeper” and perform other roles, more so by being referred to as partner organization staff by the community, it may be high time for researchers, implementers and other actors to advocate for recognition of CACs as a veritable community structure strategic to local governance, especially in service provision and so determine adequate compensation from the local government.

## Data availability statement

The raw data supporting the conclusions of this article will be made available by the authors, without undue reservation. The request can be done through https://aphrc.org/microdata-portal/.

## Author contributions

Conceptualization: CK, AN, IC, and BM. Data curation, formal analysis, and first draft writing: IC. Methodology: IC, CK, and BM. First draft review: BM, CK, CO, and CA. Final draft review: IC, BM, CK, and CO. All authors contributed to the article and approved the submitted version.
